# One-Year Outcome Predictors of Strabismus Surgery from Anterior Segment Optical Coherence Tomography with Multiple B-Scan Averaging

**DOI:** 10.1038/s41598-019-39361-5

**Published:** 2019-02-21

**Authors:** Manabu Miyata, Kenji Suda, Akihito Uji, Masayuki Hata, Akio Oishi, Eri Nakano, Akinari Yamamoto, Shinya Nakao, Hiroshi Ohtsuki, Akitaka Tsujikawa

**Affiliations:** 1 Department of Ophthalmology and Visual Sciences, Kyoto University Graduate School of Medicine, Kyoto, Japan; 2 Department of Ophthalmology, Okayama University Graduate School of Medicine, Dentistry, and Pharmaceutical Sciences, Okayama, Japan

## Abstract

Strabismologists are eager to identify preoperative or intraoperative strabismus surgery outcome predictors because of the variable effects in each patient. Conjunctival closure position recession after rectus muscle recession is effective for correcting large angle strabismus. The elasticity of the conjunctiva and Tenon’s capsule is important for strabismus surgery management. In this longitudinal study, we evaluated the prognosis of conjunctiva and Tenon’s capsule thickness (CTT) near the limbus 1 year after strabismus surgery with a limbal conjunctival incision using swept-source anterior segment optical coherence tomography with multiple B-scan averaging. Also, we identified preoperative and/or intraoperative parameters associated with corrective effects 1 year after surgery in 15 consecutive treatment-naïve patients with exotropia or esotropia who underwent recession and resection. The 1-year CTT was greater than the preoperative CTT on the resection side (269 ± 111 vs 183 ± 53 μm, *P* < 0.001) but was smaller on the recession side (137 ± 54 vs 183 ± 71 μm, *P* = 0.02). The corrective effect of surgery (1.6 ± 0.3°/mm) was most strongly correlated with preoperative CTT on the recession side (*P* = 0.005, β = −0.73). Hence, CTT on the recession side may provide adjunctive information for strabismus treatment.

## Introduction

The variable effects of strabismus surgery in each patient have impelled strabismologists to identify preoperative or intraoperative predictors of surgery outcomes. The conjunctiva and Tenon’s capsule play an important role in the outcome after strabismus surgery; the elasticity of the conjunctiva and Tenon’s capsule may be severely impaired if an eye has been deviated in one position for a long time. This factor must be considered in the aetiology of mechanically restricted ocular motility^1^. In such patients, forced closure of the conjunctiva after the muscle has weakened will counteract the effect of muscle surgery. Therefore, the conjunctiva is closed in a recessed position relative to the limbus (bare sclera) for surgical management of strabismus with a large angle in patients with thyroid-associated ophthalmopathy^2^. Furthemore, bilateral recession of the medial rectus muscle combined with recession of the conjunctiva and Tenon’s capsule for comitant esotropia increases the potential surgical benefit beyond what is possible without recession^3^.

The advent of anterior segment optical coherence tomography (AS-OCT) has facilitated objective measurements of anterior segment parameters^4–8^. The conjunctiva and Tenon’s capsule thickness (CTT) has been measured in healthy controls^9,10^ and the distance between the insertion of the extraocular rectus muscle and the limbus can be measured before strabismus surgery^11–13^. However, the image quality was poor because the device used did not allow swept-source OCT and averaging was not performed in most of the previous reports. Spectral-domain OCT cannot measure CTT in the presence of severe chemosis, which might occur immediately after muscle resection due to low penetration. Swept-source OCT (commercially available for fundus) with multiple B-scan averaging helps to provide high-quality images of the anterior sclera in patients with anterior scleral inflammation and in healthy controls^14^.

In this study, we evaluated the prognosis of CTT near the limbus 1 year after strabismus surgery with a limbal conjunctival incision, which is a widely performed procedure^15^, using swept-source AS-OCT with multiple B-scan averaging. The aim of the study was to identify preoperative and/or intraoperative parameters associated with the corrective effect of recession and resection 1 year after surgery.

## Results

After excluding 5 patients who underwent single-muscle surgery, 5 who had a small incision, 4 who were lost during follow-up, and 2 who had previously undergone strabismus surgery, 15 patients were included in our study (Table 1). One patient had undergone strabismus surgery in the opposite eye previously, and the other 14 patients analysed in the present study underwent surgery in their non-fixation eyes. The average patient age was 53.4 ± 22.1 years. Averaged temporal and nasal CTT 1, 2, and 3 mm from the limbus were not significantly different (184 ± 63 μm and 182 ± 61 μm, respectively; *P* = 0.93). Ten patients (67%) had exotropia and 2 (13%) had sensory strabismus. The amount of surgical recession and resection required was 6.3 ± 1.6 mm and 6.7 ± 1.5, respectively (*P* = 0.07). The distance between the limbus and muscle insertion site, measured intraoperatively, was 5.5 ± 0.9 mm and 5.2 ± 1.1 mm on the recession and resection sides, respectively (*P* = 0.54). The measurement site 3 mm from the limbus, was at least 1 mm from the muscle insertion site in all patients; therefore, CTT would not include muscle elements in the present study. The corrective effect was 1.6 ± 0.3°/mm and motor success was achieved by surgery in all 13 patients with non-sensory strabismus.

**Table 1:** Characteristics of the Study Population.

Clinical Parameters	Recession Side	Resection Side	*P*-value
N	15		
Age, years	53.4 ± 22.1		
Female sex, N	3		
Exotropia/esotropia, N	10/5		
Preoperative deviation, degrees	21.5 ± 4.4		
Distance between limbus and muscle insertion, mm	5.5 ± 0.9	5.2 ± 1.1	0.54
Tendon width, mm	9.4 ± 1.5	9.5 ± 1.3	0.75
Amount of surgical recession or resection, mm	6.3 ± 1.6	6.7 ± 1.5	0.07
Corrective effect, degrees/mm	1.6 ± 0.3		
One-year motor success rate (≤10 prism dioptres), %	100%		

The data are presented as the mean ± standard deviation where applicable.

The distance between the limbus and muscle insertion as well as the tendon width were measured intraoperatively using a calliper.

Two patients with sensory strabismus were excluded from the assessment of motor success.

*Statistical significance (*P* < 0.05, paired *t*-test).

### Conjunctiva and Tenon’s Capsule Thickness and Scleral Thickness

**Figure 1:**
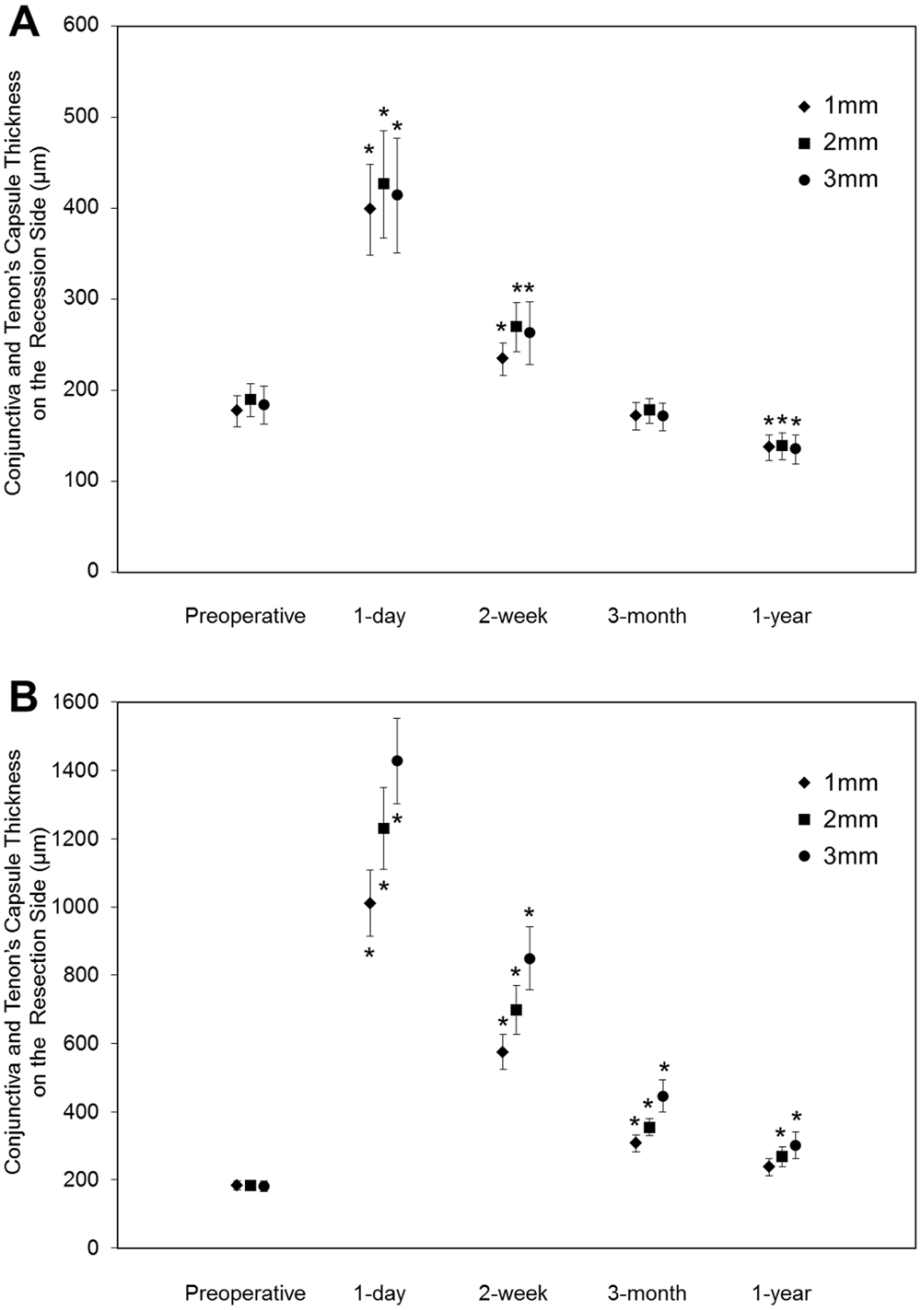
Changes in conjunctiva and Tenon’s capsule thickness on the recession (**A**) and resection (**B**) sides in the year following strabismus surgery. (**A**) The 1-day and 2-week conjunctiva and Tenon’s capsule thickness values on the recession side at 1, 2, and 3 mm from the limbus were greater and the 1-year values at 1, 2, and 3 mm were lower than the preoperative values. (**B**) Postoperative conjunctiva and Tenon’s capsule thickness measurements on the resection side were greater than preoperative measurements except for the 1-year thickness at 1 mm. The tendency for a greater thickness at 3 mm than at 1 mm on the resection side gradually resolved by the end of the first postoperative year. This tendency was not observed on the recession side immediately after surgery. The bars represent standard errors and the asterisks indicate a significant difference from the preoperative thickness.

**Figure 2:**
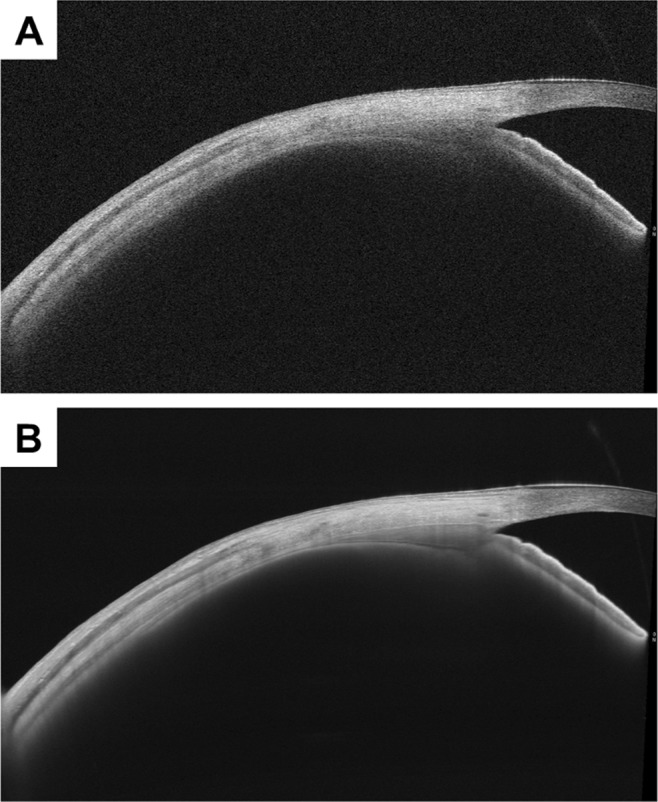
Representative images acquired via swept-source anterior segment optical coherence tomography with and without averaging. Representative images for the right eye of a 61-year-old woman without averaging (**A**) with averaging for 100 slices (**B**). The anterior segment optical coherence tomography images with averaging were acquired using originally developed software that facilitated more accurate measurements of anterior segment parameters because of its high contrast and its ability to detect fine differences between each type of tissue.

There was no significant difference in the preoperative CTT value between the medial and lateral rectus muscle sides (1 mm, *P* = 0.72; 2 mm, *P* = 0.45; 3 mm, *P* = 0.94). The postoperative CTT was greater on the resection side than on the recession side at all observation time points (Table 2). On the recession side, the CTT values at 1, 2, and 3 mm were significantly greater when measured both 1 day and 2 weeks after surgery, but smaller 1 year after surgery when compared to preoperative measurements (Fig. 1). On the resection side, the postoperative CTT values were greater than the preoperative thickness except 1 mm from the limbus 1 year after surgery. The tendency for the CTT on the resection side to be greater at 3 mm than 1 mm from the limbus gradually resolved in the year following surgery. The same tendency was not observed on the recession side immediately after surgery. There was no statistically significant change in the scleral thickness from the preoperative value between the recession and resection sides in the year following surgery, though B-scan averaged AS-OCT images enabled the detection of subtle differences between the tissues and accurate CTT measurements (Fig. 2).

**Table 2:** Conjunctiva and Tenon’s Capsule Thickness and Scleral Thickness Measured Using Anterior Segment Optical Coherence Tomography.

	Time Point of Measurement	Distance from the Limbus	Recession Side	Resection Side	*P*-value
Conjunctival and Tenon’s capsule thickness, μm	Preoperative	1 mm	177 ± 66	183 ± 51	0.79
		2 mm	189 ± 69	184 ± 55	0.80
		3 mm	184 ± 80	181 ± 59	0.92
	1 day	1 mm	398 ± 193	1011 ± 378	<0.001*
		2 mm	426 ± 228	1230 ± 466	<0.001*
		3 mm	414 ± 243	1428 ± 487	<0.001*
	2 weeks	1 mm	234 ± 70	574 ± 197	<0.001*
		2 mm	269 ± 105	698 ± 279	<0.001*
		3 mm	263 ± 134	849 ± 356	<0.001*
	3 months	1 mm	171 ± 58	307 ± 95	0.001*
		2 mm	177 ± 52	355 ± 95	<0.001*
		3 mm	171 ± 59	446 ± 180	<0.001*
	1 year	1 mm	137 ± 57	237 ± 95	0.001*
		2 mm	139 ± 57	268 ± 112	0.001*
		3 mm	135 ± 62	301 ± 152	0.002*
Scleral thickness, μm	Preoperative	3 mm	618 ± 57	616 ± 80	0.91
	1 year	3 mm	596 ± 44	630 ± 81	0.07

The data are presented as the mean ± standard deviation where applicable.

*Statistical significance (*P* < 0.05, paired *t*-test).

After we excluded 1 patient who previously underwent surgery on the opposite eye, there were no significant differences in the average CTT between non-fixation (surgery) and fixation (non-surgery) eyes (recession side, 176 ± 67 μm vs 178 ± 52 μm, *P* = 0.81; resection side, 186 ± 53 μm vs 185 ± 0.52 μm, *P* = 0.96).

### Correlation Analyses

Univariate correlation analysis of preoperative and intraoperative parameters revealed that the preoperative CTT on the recession side, the scleral thickness values on the recession and resection sides, and the distance between the limbus and the muscle insertion site on the resection side were correlated with a corrective effect with statistical significance (*P* = 0.003, r = −0.76; *P* = 0.04, r = 0.57; *P* = 0.01, r = 0.67; *P* = 0.02, r = −0.62, respectively, Table 3). Multivariate correlation analysis revealed a strong and statistically significant correlation between the preoperative CTT on the recession side and a corrective effect (*P* = 0.005, β = −0.73).

**Table 3:** Correlation Between the Corrective Effect of Strabismus Surgery and Preoperative and Intraoperative Parameters.

Clinical Parameters		Univariate Analysis		Multivariate Analysis	
		*P*	r	*P*	β
Preoperative conjunctiva and Tenon’s capsule thickness	Recession side	0.003*	−0.76	0.005*	−0.73
	Resection side	0.96			
Preoperative scleral thickness	Recession side	0.04*	0.57	0.31	
	Resection side	0.01*	0.67	0.14	
Age		0.18			
Sex (1, male; 2, female)		0.11			
Type of strabismus (1, exotropia; 2, exotropia)		0.47			
Preoperative deviation (degrees)		0.49			
Distance between limbus and muscle insertion site	Recession side	0.75			
	Resection side	0.02*	−0.62	0.12	
Tendon width	Recession side	0.73			
	Resection side	0.24			
Amount of surgical recession and resection		0.11			

The preoperative conjunctiva and Tenon’s capsule thickness is the average of the values at 1, 2, and 3 mm from the limbus.

The distance between the limbus and muscle insertion site and the tendon width were measured intraoperatively using a calliper.

These correlation analyses excluded two patients with sensory strabismus.

*Statistical significance (*P* < 0.05, paired *t*-test).

## Discussion

Our present findings suggest that the prognosis of CTT in the year following strabismus surgery can be evaluated objectively using swept-source AS-OCT images with a multiple B-scan averaging system as we observed its gradual resolution with this technique. Furthermore, our multivariate correlation analysis of preoperative and intraoperative parameters revealed that the preoperative CTT value on the recession side was the factor most strongly correlated with a corrective effect. Preoperative measurements of the CTT on the recession side may help to determine the amount of surgery required.

The negative correlation between the preoperative CTT as a non-muscular element on the recession side and the surgical effect in our study is unexpected, given the lack of significant correlation with the muscular elements of distance between the limbus and the muscle insertion site, tendon width, and amount of surgery performed. A previous report showed that recession of the conjunctiva (bare sclera) increases the effect of strabismus surgery that includes recession of the medial rectus muscle^3^. However, we observed no correlation between the preoperative CTT on the resection side and the surgical effect. We consider that because muscles are connected by the conjunctiva via the Tenon’s capsule, including the check ligament and intermuscular membrane, the conjunctiva and Tenon’s capsule at the recessed side would be pulled backward and that at the resected side would be pulled forward. When we close the wound at the limbus after strabismus surgery with a limbal incision, the recessed muscle would experience a forward force, weakening the effect of the recession, from the conjunctiva and Tenon’s capsule because the resistance of the muscle moving forward is weak; however, the resected muscle would not experience backward force by the conjunctiva and Tenon’s capsule because the resistance of the muscle moving backward is strong. As a result, tension on the conjunctiva is high on the recessed side but low on the resected side after strabismus surgery. Therefore, a higher CTT on the recession side would experience a stronger forward force via the limbal conjunctival closure and have a negative impact on the corrective effect. Taken together, we assert that preoperative CTT on the recessed side plays an important role in the corrective effect of strabismus surgery.

Perfect restoration of preoperative tissue architecture without the formation of scars is desirable for wound healing after strabismus surgery, but is hindered by inflammation, fibrosis, and tension. Various methods for preventing adhesion after inflammation and fibrosis between the muscle and surrounding tissues after strabismus surgery have been reported^16–18^. In the present study, limbal incisions on the recession and resection sides, which are widely performed, led to thinning and thickening of the conjunctiva and Tenon’s capsule, respectively, 1–3 mm from the limbus over a year. There are two plausible probabilities for these results. First, the conjunctiva and Tenon’s capsule at the recession and resection sides were pulled backward and forward by recessed and resected muscles, respectively, because muscles are connected by the conjunctiva via the Tenon’s capsule. The stretched conjunctiva and Tenon’s capsule at the recession side would become thinner and slackened, resulting in a thicker resected side. Second, postoperative inflammation and fibrosis are more likely to occur closer to the muscle. The CTT on the resection side 3 mm from the limbus was greater than that at 1 mm and 2 mm immediately after surgery. Thus, a minimally invasive small incision may be appropriate when aiming for perfect restoration of the conjunctiva and Tenon’s capsule near the limbus. Further research is needed to investigate the prognosis of the conjunctiva after strabismus surgery using other incision types.

In a previous report, a CTT of 313.54 ± 61.84 μm and a scleral thickness of 489.9 ± 64.35 μm were found at the site of insertion of the horizontal rectus muscle^9^, although the sites of measurement were different between that study and our present study. The preoperative CTT in our study, measured 1–3 mm from the limbus, which is relatively far from the muscle insertion site, was approximately 180 μm; the Tenon’s capsule, evaluated in that previous study, is rich in surrounding muscle, which may explain the smaller values recorded in our study. Another previous report with children and young adults indicated that averaged temporal and nasal conjunctival thicknesses 1, 2, and 3 mm from the scleral spur were similar (257 μm and 254 μm; we calculated)^19^. This finding is consistent with our results. In contrast, the preoperative scleral thickness of 617 μm measured 3 mm from the limbus in the present study was higher than that of a previous report, likely because the sclera under the muscle insertion site was thin^20^. Furthermore, changes in conjunctival-scleral thickness 3–5 months after strabismus surgery were recently reported^21^. Although this study did not separate CTT and scleral thickness, the combination of the conjunctiva and sclera tended to become thinner in the recession side and thicker in the resection side after strabismus surgery. Our results seem reasonable in comparison.

Use of B-scan averaged AS-OCT images utilising original software provided a high contrast, allowing us to detect fine differences between the tissues and measure CTT more accurately (Fig. 2). This averaging technique was originally reported for posterior segment OCT where a reduction of speckle noise by the alignment and averaging of multiple OCT scans from identical retinal locations resulted in improved imaging of the retina^22^. In the future, averaging will also become a standard technique for AS-OCT. However, the number of scans required may be burdensome for patients. Additional research is needed to determine the number of scans necessary for adequate evaluation of the anterior segment parameter with AS-OCT.

The measurement sites were set at 1, 2, and 3 mm from the limbus, whereas the distance between the limbus and the muscle insertion site was 4.0 mm or more when measured intraoperatively. Therefore, the measurements taken at 3 mm were at least 1 mm away from the actual muscle insertion site, indicating that CTT should not include muscle thickness. Postoperative improvements in CTT were more evident at 3 mm than at 1 and 2 mm on the resection side; i.e., the CTT at 3 mm is the best indicator of improvement in chemosis on the resection side. Thus, the CTT 3 mm from the limbus is ideal for chemosis evaluation involving the conjunctiva and Tenon’s capsule near the limbus.

In our experience, the conjunctiva and Tenon’s capsules in elderly patients were thinner than in young patients when performing strabismus surgery. However, in the present study, preoperative CTT was not significantly correlated with age (recession side, *P* = 0.09; resection side, *P* = 0.25). Hence, CTT is individually variable and induces variations in the corrective effect of strabismus surgery.

Also, stretching or dryness may affect CTT measurements. If stretching strongly affects CTT measurements, there would be a difference in the CTT between fixation and non-fixation eyes because conjunctiva and Tenon’s capsules of the recession side in the non-fixation eye would be more stretched in the opposite gaze position while obtaining AS-OCT images than that in the fixation eye. However, our results did not indicate such an occurrence. Furthermore, we considered that the effect of dryness during OCT image acquisition on measurement data would be minor because the time required to obtain AS-OCT images is short (about 2 seconds). Although we did not use topical anaesthesia or artificial tear drops while obtaining AS-OCT images, there were no adverse symptoms, including dryness or pain. However, the probabilities of these effects remain with this technique.

Univariate analysis also revealed the distance between the limbus and muscle insertion site on the resection side was negatively associated with corrective effect, and scleral thicknesses on both the recession and resection sides were positively associated with corrective effect. Although the underlying causes were unknown, axial length might be one of the causes. A previous report showed a negative correlation between axial length and corrective effect in surgery for exotropia and esotropia^23^. A longer axial length would induce a thinner sclera and shorter distance between the limbus and muscle insertion site. This, in turn, would result in a lower corrective effect. However, absence of a correlation between the distance on the recession side and corrective effect could not be accounted for only by the axial length. In the present study, the distance between the limbus and muscle insertion site on the lateral and medial rectus muscle sides was 6.0 ± 0.8 mm and 4.5 ± 0.5 mm, respectively; the difference was significant (*P* < 0.001). However, because of the small sample size, we included cases of esotropia and exotropia in the same analysis, which might also have affected our result. Additional studies including axial length and other potential parameters with separate analyses for cases of esotropia and exotropia are needed.

This study has several limitations, in particular its small sample size and the manual segmentation method used to measure thickness. However, it should be noted that we did not include patients aged ≤12 years, because the policy at our institution is to perform a Guyton’s small incision^24^ under general anaesthesia in these patients. Further research is needed to compare limbal incisions with small incisions in age-matched patients. Though our small sample size was small, there was no correlation between the type of strabismus and the corrective effect in our study, as demonstrated in our sub-analysis of exotropia cases (n = 9) that produced similar results (Table 4). Overall, multivariate correlation analysis revealed that the feature with the strongest correlation with the corrective effect was preoperative CTT on the recession side (*P* = 0.01, β = −0.78). Additional studies with large sample sizes and separate types of strabismus are warranted to further substantiate the results in our study. Furthermore, the manual segmentation and measurements were performed as in previous reports^9,10^. However, the development of an automatic segmentation and measurement method may reduce measurement errors further.

**Table 4:** Correlation Between the Corrective Effect of Strabismus Surgery and Preoperative and Intraoperative Parameters in Subanalysis of Exotropia (N = 9).

Clinical Parameters		Univariate Analysis		Multivariate Analysis	
		*P*	r	*P*	β
Preoperative conjunctiva and Tenon’s capsule thickness	Recession side	0.005*	−0.83	0.01*	−0.78
	Resection side	0.62			
Preoperative scleral thickness	Recession side	0.07	0.63	0.19	
	Resection side	0.7	0.63	0.45	
Age		0.29			
Sex (1, male; 2, female)		0.13			
Preoperative deviation (degrees)		0.26			
Distance between limbus and muscle insertion site	Recession side	0.14			
	Resection side	0.04*	−0.69	0.06	
Tendon width	Recession side	0.33			
	Resection side	0.52			
Amount of surgical recession and resection		0.03*	−0.72	0.24	

The preoperative conjunctiva and Tenon’s capsule thickness is the average of the values at 1, 2, and 3 mm from the limbus.

The distance between the limbus and muscle insertion site and the tendon width were measured intraoperatively using a calliper.

These correlation analyses excluded one patient with sensory exotropia.

^*^Statistical significance (*P* < 0.05, paired *t*-test).

In conclusion, the CTT did not reach the preoperative value on either the recession side or the resection side 1 year after surgery. Preoperative measurements of CTT on the recession side using AS-OCT with multiple B-scan averaging may help to determine the amount of surgery required.

## Methods

This longitudinal study was approved by the ethics committee of Kyoto University Graduate School of Medicine (Kyoto, Japan). The study protocol adhered to the tenets of the Declaration of Helsinki. All patients provided informed consent.

### Patients

Consecutive patients with exotropia or esotropia who visited the Department of Ophthalmology and Visual Science at the Kyoto University Graduate School of Medicine between July 2016 and April 2017 were recruited for the study. All patients underwent a comprehensive strabismological examination, including a prism and alternate cover or Krismky test, a Bagolini striate glasses test, a stereotest, and an eye movement test, as well as a comprehensive ophthalmological examination. Exotropia or esotropia was diagnosed by strabismological specialists. The amount of surgery required was determined by the results of the prism adaptation test.

The inclusion criteria were as follows: combination of recession and resection for the horizontal rectus muscles to correct exotropia or esotropia with limbal incision, treatment-naïve muscles in the operated eye, and at least 1 year of follow-up. Patients who underwent surgery that involved a small conjunctival incision or a single muscle were excluded. The criterion for surgical motor success was defined as horizontal deviation ≤10 prism dioptres in patients without sensory strabismus after 1 year.

### Measurement of Conjunctiva and Tenon’s Capsule Thickness and Scleral Thickness

**Figure 3:**
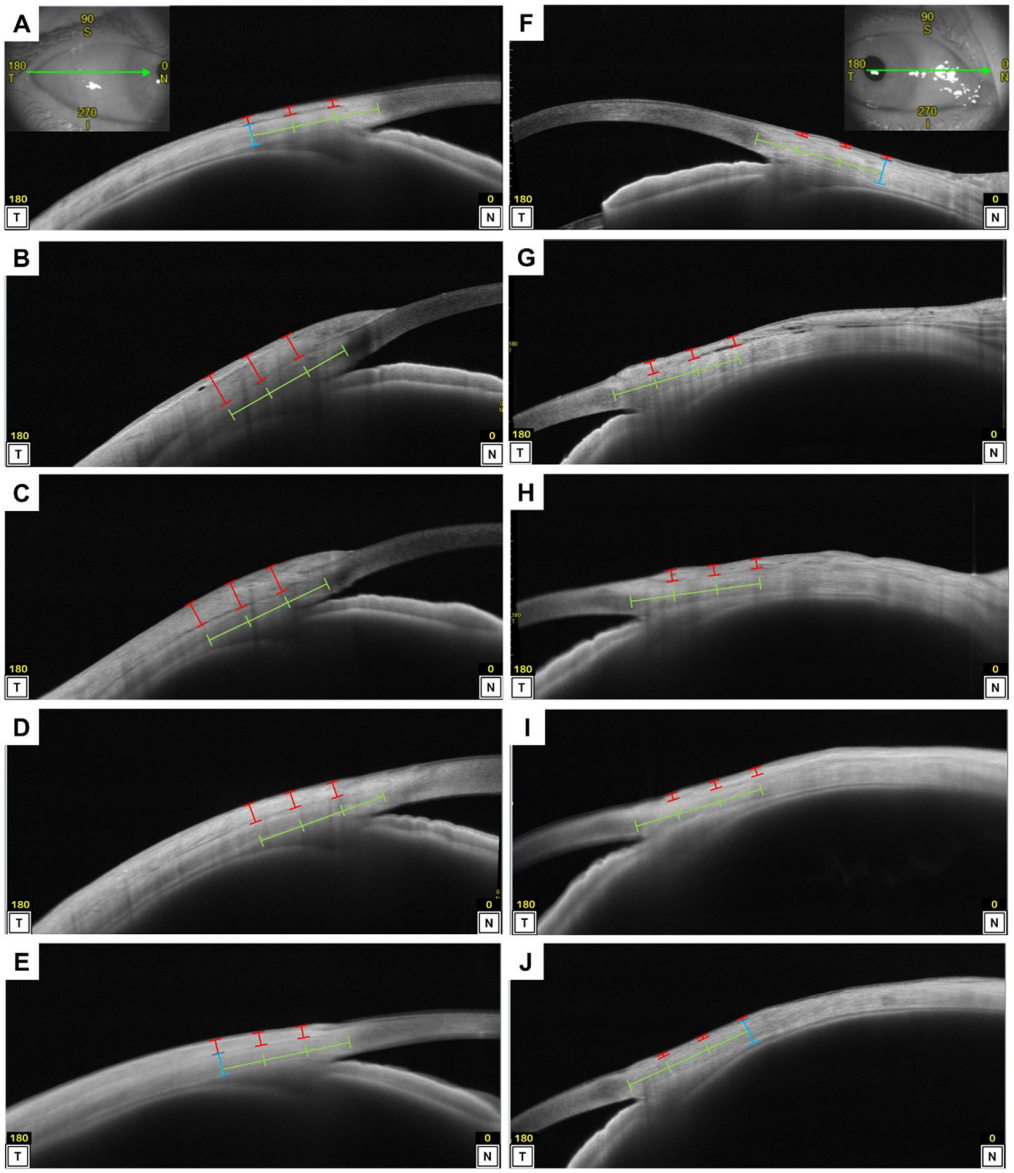
Representative anterior segment images acquired by swept-source anterior segment optical coherence tomography before and after strabismus surgery. Representative images of the right eye of a 60-year-old woman with esotropia who underwent a combination of medial rectus recession (6.0 mm) and lateral rectus resection (7.5 mm) are shown. The red bars represent the conjunctiva and Tenon’s capsule thickness. The blue bars represent the scleral thickness. The green bars represent distances of 1, 2, and 3 mm from the limbus. (**A**–**E**) Horizontal B-scan images on the lateral rectus muscle side through the pupil. (**F**–**J**) Horizontal B-scan images on the medial rectus muscle side through the pupil. Each image was obtained before surgery (**A**,**F**), and 1 day (**B**,**G**), 2 weeks (**C**,**H**), 3 months (**D**,**I**), and 1 year after surgery (**E**,**J**). The mean preoperative and 1-day, 2-week, 3-month, and 1-year postoperative thickness values on the lateral rectus muscle side were 191 μm (**A**), 700 μm (**B**), 686 μm (**C**), 399 μm (**D**), and 353 μm (**E**), respectively; the respective values on the medial rectus muscle side were 85 μm (**F**), 256 (**G**), 277 μm (**H**), 182 μm (**I**) and 88 μm (**J**).

For each patient, the eye that underwent surgery was analysed in the 3 and 9 o’clock positions. Horizontal B-scan images through the centre of the pupil were obtained using swept-source AS-OCT (CASIA2, Tomey, Nagoya, Japan) before surgery, and at 1 day, 2 weeks, 3 months, and 1 year after surgery at the lateral gaze positions and gaze positions opposite of the imaged side without the use of eye drops. This OCT device has a wavelength of 1310 nm that allows visualisation of the deep tissues and images to be acquired carefully without the patient being subjected to the discomfort of glaring. Since the AS-OCT device was not commercially equipped with a multiple B-scan averaging system, original software was developed for the device. Using this software, we were able to obtain multiple images for averaging using up to 100 slices. One investigator (MM) manually measured the CTT perpendicular to the scleral interface at 1, 2, and 3 mm from the limbus (the transition between the corneal epithelium and the conjunctival epithelium, as described elsewhere^12^), because the sites can indicate bare sclera and because CTT can be accurately measured without muscle at these sites. A built-in calliper was used in accordance with a previous report (Fig. 3)^9^. The scleral thickness 3 mm from the limbus was also measured before and 1 year after surgery.

### Surgical Procedures and Intraoperative Measurements of the Distance Between the Limbus and Muscle Insertion and Tendon Width

One surgeon (MM) performed a limbal incision and two 3–4 mm radial incisions^1^ after applying 4% lidocaine eye drops (Xylocaine Ophthalmic Solution 4%, Aspen Japan, Tokyo, Japan) and eye drops containing 1% lidocaine with 0.001% epinephrine (Xylocaine Injection 1% with Epinephrine, Aspen Japan). SubTenon’s injections of 2% lidocaine (Xylocaine Injection Polyamp 2%, Aspen Japan) were performed. The intermuscular membrane and check ligament were cut, and the rectus muscle was freed. Recession or resection was then performed using a muscle clamp fixation with 2 or 3 knots of 7-0 nylon (7-0 Ortho suture, Handaya, Tokyo, Japan), respectively. The surgeon measured the distance between the limbus and the centre of the muscle insertion as well as the tendon width at the level of insertion in 0.1 mm steps using a Castroviejo calliper. The remainder of the muscle insertion tendon on the recession side was cut to flatten the scleral interface. Finally, the conjunctival wound was closed at the limbus (no bare sclera) with 4 knots of 8-0 virgin silk. Cautery was used intraoperatively for hemostasis. After surgery, all patients used 0.1% fluorometholone (Flumetholon Ophthalmic Suspension 0.1%, Santen, Osaka, Japan) and 0.3% gatifloxacin (Gathiflo Ophthalmic Solution 0.3%, Senju, Osaka, Japan) eye drops for 2–4 weeks.

### Statistical analysis

The data are presented as the mean ± standard deviation where applicable. The preoperative and 1-year horizontal deviation values were obtained from the results of prism adaptation testing and prism and alternate cover testing, respectively. Prism dioptres were converted to degrees for the statistical analyses. The corrective effect of recession and resection (°/mm) was defined as the difference between the 1-year and preoperative deviation (°) per sum of recession and resection distance (mm) as described previously^25^. Comparative analyses were performed using paired *t*-tests. Correlation analyses were performed using Spearman’s rank correlation coefficients. In the correlation analysis, the average CTT measured 1, 2, and 3 mm from the limbus were taken as the representative values. Multiple stepwise regression analysis was performed using the corrective effect (except in patients with sensory strabismus) as the dependent variable and preoperative or intraoperative parameters with Spearman’s correlation coefficients and *P*-values < 0.10 as independent variables. All statistical analyses were performed using SPSS software (version 21, IBM Corp., Armonk, NY). *P*-values < 0.05 were considered statistically significant.

## Data Availability

All data generated or analysed during this study are included in this published article.
